# The 2025 Iberian Blackout: Will There be More?

**DOI:** 10.1007/s40518-026-00297-w

**Published:** 2026-09-15

**Authors:** Janusz W. Bialek

**Affiliations:** School of Electrical and Electronic Engineering, Imperial College London, Exhibition Road, South Kensington, London, SW7 2AZ UK

**Keywords:** Power system blackouts, Security of supply, Iberian blackout 2025

## Abstract

**Purpose of Review:**

The goal of the paper is to analyse the Iberian blackout and determine its direct and underlying causes. We ask the questions: Are renewables to blame? Are other systems with high renewable penetration in danger of a blackout?

**Recent Findings:**

There are few publications analysing the blackout. Only one discusses lessons for modern power systems and policy implications.

**Summary:**

The direct cause of the Iberian blackout was an unexpected interaction between oscillations and voltage stability compounded by inadequate voltage control arrangements in Spain. However, the underlying cause is that Spain has concentrated in recent years on increasing the share of renewables but seems to have paid less attention to adapting power system operation and control to the changing needs. The rapidly increasing share of renewables in many power systems causes a profound change in the way power systems behave, which we do not yet fully understand. Hence a significant interdisciplinary effort of academia and industry is required to overcome the challenges and prevent future blackouts.

## Introduction

The Iberian blackout of April 28, 2025, was the largest blackout in Europe since the 2003 Italian blackout. 55 million of people in Spain and Portugal, and a little bit of France, lost supply. As renewable energy resources (RES) in Spain and Portugal form a significant part of overall generation, people were asking questions. Are renewables to blame? Are other systems with high RES penetration in danger of a blackout? This article will attempt to answer those questions.

As the direct reason for the blackout was quite technical (interactions between power oscillations and voltage stability), we start from a short overview of what reactive power is and its link to voltages, describe the principles of voltage control in power systems and what power oscillations are and how they can be damped. Then we proceed with a description of the events and discuss the direct and underlying institutional reasons for the blackout. We finish with recommendations on how to avoid future blackouts.

This article is based on the information provided by the Spanish Transmission System Operator Red Electrica de Espana (REE) [1], the Spanish government report [2] and two reports by ENTSOE [3, 4]. There are few publications analysing the blackout due to delays in publications of ENTSOE reports. Among them [5] discusses lessons for modern power systems and policy implications while [6] presents technical analysis of the voltage stability.

## Background Information on Reactive Power, Voltage Control and Oscillations

This section contains a background technical information on reactive power, voltage control and power system oscillations necessary to understand how the blackout happened.

### Reactive Power

First let us explain what reactive power is as it is an elusive and not well-understood concept. Reactive power (usually denoted by Q) is a phenomenon connected with the existence of inductors and capacitors in AC circuits. There is no reactive power in DC circuits. It is important to appreciate that inductors (coils, reactors) and capacitors (condensers) store energy: an inductor stores magnetic energy proportionally to the square of the current flowing through it while a capacitor stores electric energy proportionally to the square of the voltage across it.

Now let us consider an inductor supplied by an AC source. The AC current magnetises and demagnetises the inductor twice in every AC cycle (also in the negative half-cycle as magnetic energy is proportional to the square of the current) so energy flows back and forth with double the fundamental frequency (100 Hz = 2 × 50 Hz in Europe). Reactive power quantifies this oscillating energy and its unit is VAR. On average (i.e., over the cycle) the reactive energy flow is zero (which means that it cannot be used to do any useful work) –but by convention we say that reactors “consume” reactive power.

The situation is similar but opposite with the capacitors. The AC voltage oscillates across the capacitor which means it is charged/discharged twice in every cycle. Hence the (electric) energy again flows back and forth with 100 Hz frequency, but the exchanges are 180^0^ out of phase (i.e., in anti-phase) with respect to those in an inductor. This means that *an inductor and a capacitor perfectly compensate each other*: when one needs energy, the other gets rid of it. By convention, we say that capacitors “produce” reactive power.

The most important effect of reactive power is its influence on network voltages. It takes some circuit theory, which we will skip, to prove that *inductive reactive power causes voltages to drop* while *capacitive reactive power causes voltages to rise*. Hence controlling reactive power production/consumption is essential for voltage control.

### Voltage Control

Voltages in a grid have to be kept within tight limits, usually less than 10% of their nominal values.

The main means for controlling voltage in a power system are *synchronous machines* operating at traditional thermal, nuclear or hydro power stations. They can produce or consume reactive power smoothly (dynamically) by controlling the DC excitation (rotor) current^1^. The other means of voltage control are static (i.e., not rotating) devices, which can control reactive power in steps, such as *reactors* (i.e., inductors absorbing reactive power and therefore lowering voltage), *capacitors* (producing reactive power and therefore increasing voltage) or *Static Var Compensators (SVC)* which contain a combination of inductors (reactors) and capacitors switched on and off by power electronics as required. Loads are typically inductive and therefore lower the voltage. *Transmission lines* can be sources or sinks of reactive power depending on the load current flowing through them. If the current is high (i.e., during the day), the inductive effect prevails so the line lowers the voltage. If the current is low (i.e., during the night), the capacitive effect prevails and the line increases the voltage. Hence System Operators often disconnect some lines when system voltages are high, as increasing the load on the remaining lines increases the inductive effect and lowers voltages.

Additional means of voltage control are *tap-changing transformers* connecting high-voltage transmission to lower-voltage distribution networks. As most of electrical loads are connected to distribution networks, maintaining the distribution voltage within the limits is of utmost importance. When voltage at the distribution level is too low or too high, the tap changer changes the transformation ratio hence changing the secondary (distribution) voltage. Tap changers are mechanical devices which means that they are slow and cannot be used too often to prevent wear and tear.

RES such as wind and solar PV are connected to the grid by means of power electronics devices called *inverters*^2^ – hence they are often called Inverter-Based Resources (IBRs). Inverters can produce or absorb reactive power (within limits) so they can be utilised for voltage control. Hence many power systems with high penetration of RES, such as e.g., Great Britain (GB) or Portugal, utilise RES for voltage control but, crucially, not Spain which was one of the major contributing factors to the blackout. In Spain, RES were asked to operate at a constant power factor, i.e., with reactive power output (usually inductive) depending just on the real power output, rather than depending on the voltage, which meant they did not participate in voltage control. In Portugal, RES plants with output higher than 1 MW do contribute to voltage control. Hence Portugal did not experience voltage problems but was brought down by Spain.

The Iberian blackout was the first major blackout which was caused by overvoltage, i.e., voltages raising above their nominal levels. Usually voltage stability is analysed from the point of view of undervoltage, i.e., voltages dropping below their nominal levels, which tended to be seen as the main danger to system security. For example RES plants are required to follow Fault Ride Through (FRT) characteristics which ensure that they remain connected for a certain time to the grid when voltages drop suddenly due to faults (short-circuits). Problems with overvoltages did not attract a similar research attention so far as undervoltage. This is likely to change following the Iberian blackout.

### Power System Oscillations

Oscillations are a routine occurrence in power systems and are usually caused by an event, e.g., disconnection of a line or tripping of a large power station, but they may also occur spontaneously, i.e., without any apparent reason, caused by unexpected control interactions between generating units or a resonance between grid elements. *Electromechanical oscillations* have a spring-like character and are caused by rotors of synchronous machines swinging against each other. It means that their speed oscillates slightly, with the frequency of oscillations less than 1 Hz, around the nominal speed (50 Hz in Europe). The rotor speed oscillations cause frequency oscillations, as grid frequency is determined by the rotor speed. They also result in large power transfer oscillations due to large masses of generators involved so they have to be quickly damped. Spontaneous electromechanical oscillations tend to happen when there is a weak network connection between generators and power transfers are high.

There are a number of well-known modes of oscillation in Europe whereby rotors of synchronous machines in a country, or a group of countries, start to swing spontaneously against another group – they are referred to as inter-area oscillations. Hence many synchronous generators at strategic locations are equipped with *power system stabilisers* (PSS) which are additional controls increasing damping of power swings. Similarly HVDC converter stations are equipped with *power oscillation dampers* (PODs).

The mechanism of inter-area oscillations is well-known, and System Operators know how to deal with them. The measures include reducing inter-area power flows, strengthening the network by re-connecting previously disconnected transmission lines and increasing the network voltage.

Over the last 10 years or so a new type of oscillations, dubbed *Sub Synchronous Oscillations* (SSOs), has been observed in many grids with a high penetration of IBRs. Inverters are programmable devices and they have a high number of control loops ensuring their required behaviour. A behaviour of a grid with a high penetration of IBRs is still not well understood and sometimes the controllers of inverters may misbehave causing spontaneous SSO for no apparent reasons. This problem is the subject of intensive research. Note that the means of dealing with inter-area oscillations, described in the paragraph above, may not be effective in damping SSOs as their mechanism is completely different.

## Description of Events

The day of the incident was a typical spring day in Spain, with mild temperatures and mostly sunny weather. The system’s solar generation was similar to previous days, while wind generation was more variable but within the ranges observed in previous days. About 70% of generation in Spain was produced by wind and solar PV (mainly in south-west of the country), with exports to France, Portugal and Marocco. One of the main well-known possible problems in systems with high RES is low inertia^3^, but in Spain inertia was adequate and played no role in the blackout. Voltages were within the limits. However only 10 traditional power stations provided voltage support, which was fewer than normal due to fire in one of the plants. An additional plant was instructed to connect but did not manage to do so in time before the blackout. Importantly, few traditional plants providing voltage support were located in the south-west of the country, where the majority of RES were installed. This was potentially a problem as voltage support is local in character.

### Oscillations

The grid started to experience two periods of voltage, frequency and power oscillations about ½ hour before the blackout. Although the oscillations did not directly cause the blackout, they were a significant contributing factor.

The first period of oscillations started at 12:03 and lasted about 5 min. The frequency of oscillations was 0.63 Hz and it was a local Iberian oscillation that did not propagate to the rest of Europe. Subsequent analysis revealed that it was a so-called forced mode of oscillations (i.e., SSO) caused by controls of a RES plant (not named in the reports).

The second period, with the frequency of oscillations of 0.21 Hz, started at 12:19 and lasted about 3 min. This mode of oscillations was a well-known East-West mode whereby generators in Iberia swing against Eastern Europe (Baltics, Hungary, Poland, and Turkey).

To deal with the oscillations the Spanish System Operator, Red Eléctrica de España (REE), undertook the following actions:

- 10 previously disconnected transmission lines were reconnected to strengthen the network. This had a side-effect of reducing flows on other lines, as some of their load was carried now by the newly connected lines. This reduced their inductive effect, and therefore caused an increase in system voltages.
- Exports to France were reduced by reducing output of some of the southern plants. This had again a side-effect of reducing power flows across Spain and therefore increasing voltages.
- Some reactors were disconnected to increase the voltages and improve damping of oscillations.
- The mode of operation of the HVDC link to France was changed from AC emulation to constant power^4^. This means that power was still exported even after generation trips in Spain started causing power deficit. Changing the mode of operation did not have a direct effect on the voltages.

The measures undertaken to damp oscillations had a minor influence on damping of the 0.63 Hz forced mode but were effective in damping of the 0.21 Hz inter-area mode. Post-mortem analysis showed that PSS on some of large units in Spain were missing and insufficient actions were undertaken by the existing ones.

### Generation Trips

The measures undertaken to damp oscillations caused a significant increase in system voltages and led to plants, mainly RES, tripping on overvoltage as described in the rest of this section. This caused a vicious circle: tripping of a plant causes a reduction of power flows and therefore reduction of the inductive effect of transmission lines and an increase in voltage. Additionally, as those plants operated at a constant inductive power factor, their tripping removed reactive power absorbed by the plants. This led to further increase in voltage and subsequent trips.

**Fig. 1:**
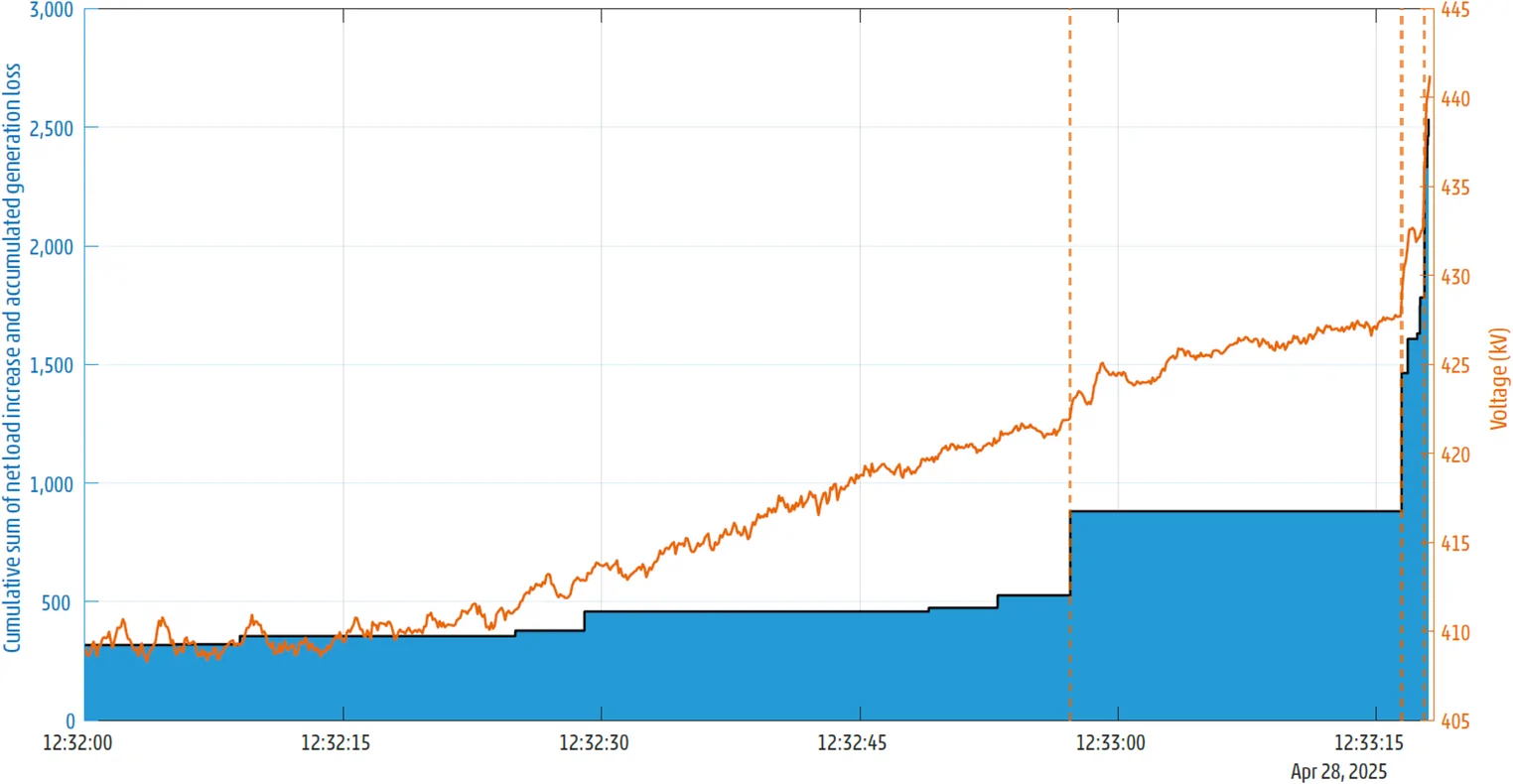
Cumulative power lost and load increase (in blue) and voltage (in orange) in the Iberian peninsula within the 12:32:00–12:33:18 time frame. Vertical dashed lines indicate the first three stages of the blackout

Figure 1 shows the cumulative power imbalance (the sum of generation lost and demand increase) and the voltage at one of the transmission substations within the 12:32:00–12:33:18 time frame. Before the trips started, there were no oscillations and transmission voltages were within the limits.

#### First Stage: 12:32:00–12:32:57

The first stage started with the power output of large RES generators (higher than 5 MW) in Spain decreasing by approximately 500 MW between 12:32:00 and 12:32:48. Between 12:32:00 and 12:32:57, 208 MW of distributed wind and solar generating units in northern and southern Spain tripped due to unknown reasons. In the same period, there was an increase in net load in the distribution grids of approximately 317 MW, which can be attributed partly to disconnections of small distribution-connected generators < 1 MW (mainly rooftop PV) and partly to an increase in voltage-dependent loads as a result of a voltage rise. The exact reasons for some of these events are not known.

#### Second Stage: 12:32:57 − 12:33:17

From around 12:32:20, a particularly steep rise in local voltage occurred at a substation in the area of Granada. This initiated the second stage starting at 12:32:57 when a 400/220 kV transformer in that substation tripped due to the activation of an overvoltage protection disconnecting several PV, wind and thermo-solar plants, resulting in the loss of 355 MW, and an additional step change in voltage.

The next event consisted of two sets of trips, resulting in an additional loss of 727 MW of PV and thermo-solar facilities connected to two 400 kV transmission substations which tripped on overvoltage.

#### Third Stage: 12:33:17 − 12:33:18

The third stage lasted just one second when several trips occurred leading to the disconnection of wind and solar generation of 928 MW. Some of these trips occurred due to overvoltage protection, but the ENTSOE Expert Panel was not able to establish the cause of most of these trips. The post-mortem analysis indicated that the overvoltage protection settings at some generation units were set below the voltage limits established in accordance with the applicable requirements.

Note that transmission voltages until 12:33:18 in Spain were lower than the allowed maximum of 435 kV, yet power plants were tripping. No generation trips were observed in Portugal or France within the 12:32:00–12:33:18 timeframe.

#### The Final Phase of the Blackout: 12: 33:18 − 12:33:24

Between 12:33:18 and 12:33:21, the voltage in the South of Spain increased sharply, and consequently also in Portugal. The overvoltage triggered a cascade of generation losses, causing the frequency of the Spanish and Portuguese power systems to decline. At 12:33:19, the power systems of Spain and Portugal started losing synchronism with the rest of the European system. Between 12:33:19 and 12:33:22, the automatic load shedding^5^ of Spain and Portugal was activated, but was unable to prevent the collapse of the Iberian power system as the core reason was overvoltage rather than deficit of generation. Load shedding actually made the overvoltage worse as the reactive power-absorbing loads were disconnected and line loadings also decreased hence further increasing voltages. However the subsequent investigation revealed that blocking load shedding would not have prevented the blackout.

At 12:33:20.473, the AC interconnection to Morocco tripped due to underfrequency in Spain. At 12:33:21.535, the AC overhead lines between France and Spain were disconnected by protection devices due to a loss of synchronism, preventing the propagation of the perturbation into Europe.

Finally, at 12:33:23.960, the electrical separation of the Iberian system was completed by tripping the HVDC lines that were still transmitting power from Spain to France (due to the constant power mode setting at the time). The total blackout occurred less than 1.5 min after the first trips.

Restoration of supplies was relatively quick. The Spanish system was fully restored within 16 h, while the Portuguese system was back online within 12 h after the blackout, with many parts regaining power much quicker.

## Why Voltage Control was Slow and Ineffective

An obvious question arises why REE was unable to contain the fast rise in voltages. The main reasons were [4]:

- There were only 10 conventional plants available able to undertake dynamic voltage control. Recall that synchronous generators at traditional plants are the main means of voltage control.
- A number of conventional plants did not control voltage effectively as they delivered less than 75% of reactive power absorption demanded. One of the reasons for this non-compliance was that there are no penalties for non-delivery of reactive power in Spain.
- RES plants in Spain operated at a constant power factor so could not provide a flexible voltage support. Since 2020, new RES plants were required to be capable to participate in voltage control but that functionality was not activated at the time as it was a new, untested feature. Following the blackout, an increasing number of RES plants started to participate in voltage control.
- Tripping of RES plants operating at constant power factor led to a fast reduction of reactive power absorption and also a fast increase in voltage.
- Tap-changing transformers operate with dead-zones and delays to avoid unnecessary operation so they could not react quickly to voltages increasing quickly.
- Shunt reactors are manually operated in Spain so again they could not be quickly reconnected. Note that in France about half of reactors can be automatically connected if voltage rises quickly.
- Design of voltage control of local generation networks (behind the connection point) was poor and not aligned with system needs.
- Many overvoltage disconnection protection settings diverged from applicable requirements or were not aligned with system needs.
- Spanish 400 kV grid is operated at a wider voltage range than in other EU countries, enabled by specific provisions applicable to Spain.

Due to the perceived shortcomings in voltage control, in April 2026 the National Markets and Competition Commission (CNMC) opened a probe into REE and power companies [7]. The CNMC has identified various indications of non-compliance that may have affected the operation of the electricity system and could constitute administrative offences. CNMC was probing REE over “very serious” alleged breaches of Spain’s electricity law, pointing to sections on the scheduling of power generation, the balancing of the grid system, information sharing and the issuance of instructions to power suppliers. The CNMC opened five probes apiece into the big power generators — Iberdrola, Naturgy and Endesa — and one into Repsol, saying it had found indications of “serious” breaches of the law.

## The Underlying Institutional Reasons for the Blackout

While the direct reasons for the blackout were inadequate voltage control arrangements in Spain, the underlying institutional reason are much deeper. Spain has some of the most ambitious renewable energy targets in Europe so, in order to achieve them, the country has concentrated in recent years on increasing the share of RES but seems to have paid less attention to adapting power system operation and control to the new conditions. One cannot run a system with a high penetration of RES the same way as a conventional system. Many other countries with a high penetration of RES, notably GB, Ireland and Portugal, seem to have done a much better job in that respect - see for example [8]. Most relevantly to the blackout, Spain did not employ IBRs for voltage control, despite obvious benefits of that.

After the blackout, REE warned about voltage instability linked to renewables and urged technical changes. In October 2025, the system operator began implementing the framework and authorising renewable plants to provide voltage control. By 2026, additional rules were adopted to further strengthen voltage control and grid stability [9].

## Are Future Similar Blackouts Inevitable?

Does it all mean that the Iberian blackout was a particular Spanish mishap and the rest of the world can sleep in peace? Unfortunately, it does not seem to be the case. Fast replacement (in many parts of the world) of gas/coal power stations, which operate with synchronous machines, by RES (connected to the grid by inverters) changes fundamental characteristics of the system. They are no longer determined by the physics of synchronous machines (and physics never fails) but by software of inverters (which may cause unexpected and potentially harmful effects). System operation, control and planning have to be quickly adapted to the changed system profiles.

Note that it took about 100 years, from the end of the 19th Century, for traditional power systems to mature. Power systems did change during that period but the changes were slow so there was time to adapt. But now the pace of change is much faster. We must make in about 10 years the equivalent of 100 years of progress in understanding power systems as more and more RES are commissioned. A significant research effort is needed as there are many problems, apart from SSO, which we simply do not know how the solve and also there are many problems we do not know about yet – it is a problem of so-called “unknown unknowns”. The necessary research must be multi-disciplinary as it requires close cooperation between power system engineers, power electronics experts (to deal with the design of inverters), control theory experts (to design control systems of inverters), data scientists (as many problems are difficult to solve analytically) and economists (as new services need to be designed). It requires a combined effort of the industry and academia to overcome the challenges. An example of such efforts is the Power System Transformation Consortium (GPST) which published in 2025 a modified Research Agenda [10] with 48 IBR-related research questions which require urgent answers. It should be emphasised that the problems are not unsurmountable, they just require a significant research effort to solve. In most cases, no hardware investment is needed – just research. As we do not know yet the answers to many of the questions, do not be surprised if you hear about another blackout soon.

## Conclusions

The direct cause of the Iberian blackout was an unexpected interaction between oscillations and voltage stability compounded by inadequate voltage control arrangements. Actions taken to damp oscillations had a side effect of increasing suddenly the voltages in the Spanish network which caused some plants to trip. This caused a vicious circle: tripping of plants caused voltages to rise even more due to reduced line loading and lost reactive power consumption, which finally led to the full blackout.

However the underlying causes were much deeper and institutional in nature. Spain has concentrated in recent years on increasing the share of RES but seems to have paid less attention to adapting power system operation and control to the changing needs. One cannot run a system with a high penetration of RES the same way as a conventional system.

Power systems are changing quickly with rapidly increasing share of RES in many parts of the world. This causes a profound change in the way power systems behave, which we do not yet fully understand. Hence a significant concerted research effort of industry and academia is required to overcome the challenges and prevent future blackouts.

## Key References

- ENTSOE: “Grid Incident in Spain and Portugal on 28 April 2025. ICS Investigation Expert Panel Final Report”, 20 March 2026.
  - ○  This is the main authoritative report on the blackout and the source of most of information in the paper.
- Hugo Morão: “The 2025 Iberian Peninsula blackout: Lessons for modern power systems and policy implications”, Utilities Policy 101 (2026).
  - ○  This paper has been written after ENTSOE has published its Factual Report [3] but before publishing the Final Report [4]. It contains an analysis of the blackout and suggests policy implications.
- L. Rouco, F.M. Echavarren, E. Lobato: “The overvoltage-driven blackout of the Iberian Peninsula on 28th April 2025”, Sustainable Energy, Grids and Networks, March 2026.
  - ○  This paper contains a technical voltage stability analysis of the blackout. An illustrative 3-bus example is used and shows that inadequate safety margins led to the blackout.

## Data Availability

No datasets were generated or analysed during the current study.

## Footnotes

1 This explains why a synchronous machine which is freely-rotating (i.e., not connected to a turbine) is called a *synchronous condenser (*or *compensator***)** as it can be used for reactive power compensation (and also to provide inertia).

2 Alternatively the term *converters* is often used.

3 Inertia, which is provided by large rotors of synchronous generators in traditional plants, helps maintain stability of a power system. RES provide little inertia.

4 HVDC connection separates systems from a frequency control point of view. Hence sometimes HVDC converters are programmed to behave as if they were AC lines, hence allowing countries to help each other with frequency control.

5 Load shedding, technically referred to as Low-Frequency Demand Disconnection (LFDD), is a system defence action that, when frequency drops below certain level indicating a severe power imbalance, automatically disconnects some parts of distribution networks with their loads to reduce system load and improve power balance.
